# Characteristics of healthcare workers who died during the fight against COVID-19 in China

**DOI:** 10.12669/pjms.37.1.3384

**Published:** 2021

**Authors:** Yujun Wang, Yun Ji, Yuedong Wang

**Affiliations:** 1 Yujun Wang, PhD. School of Humanities and International Education, Zhejiang University of Science and Technology, Hangzhou, China; Zhejiang Institute of New Media for Government Affairs, Hangzhou, China. Second Affiliated Hospital Zhejiang University School of Medicine, Hangzhou, China; 2 Yun Ji, MD. Department of Surgical Intensive Care Unit, Second Affiliated Hospital Zhejiang University School of Medicine, Hangzhou, China; 3 Yuedong Wang, MD. Department of General Surgery, Second Affiliated Hospital Zhejiang University School of Medicine, Hangzhou, China

**Keywords:** Characteristics, healthcare workers, Fatality, COVID-19, China

## Abstract

Coronavirus disease 2019 (COVID-19), first reported in December 2019 in Wuhan, China, has progressed to a pandemic associated with substantial morbidity and mortality. Little is known about the healthcare workers who died fighting the disease in China. This paper analyzed the data of 78 Chinese healthcare workers who died in the fight against COVID-19 between 23 January and 2 June, 2020, and revealed the following characteristics. First, compared to the number of deaths directly attributable to COVID-19, more healthcare workers died from pre-existing disease attack induced by excessive fatigue or died from accidents. Second, the median age of the healthcare workers who died directly from COVID-19 was younger than that of the Wuhan non- healthcare workers who died of COVID 19. Third, although more women than men were involved in fighting the pandemic, more men died. Fourth, more healthcare workers died in Hubei than in other provinces. Fifth, most of the healthcare workers who died directly from COVID-19 were non-professionals.

Coronavirus disease 2019 (COVID-19), first reported in December 2019 in Wuhan, Hubei province, China, has progressed to a pandemic associated with substantial morbidity and mortality.^1^ By June 13, 2020, 83132 confirmed cases had been reported in China, causing 4634 deaths.^2^ Responding to the pandemic, China focused on traditional public health outbreak response tactics-isolation, quarantine, social distancing, and community containment^3^, which have never before been implemented on such a large scale.^4^,^5^ Further, a total of 42,600 healthcare workers (28,000 women) from other provinces were sent to Hubei to fight the disease.^6^

Although the disease has been controlled, the whole country has paid a high price. Asides from more than 116.9 billion RMB (approximately16.5 billion USD) allocated for pandemic prevention and control^7^, there were public health interventions and sacrifices that cannot be estimated in monetary terms. On March 5, the Chinese government announced a commendation list of advanced collectives and individuals of the national health system who had contributed to the prevention and control of COVID-19 pandemic. A total of 506 healthcare workers were honored, but 34 of them had died.^8^ Little is known about the healthcare workers who died fighting the disease. This paper analyzed the characteristics of 78 Chinese healthcare workers who died in the fight against COVID-19.

Based on official reports from government agencies^9^ and reports from news websites, we searched for information on the deaths of 78 Chinese medical workers during the pandemic between 23 January and 2 June, 2020. The following public access data were collected: age, gender, cause of death, professional or not, and their location. Continuous variables are described as medians and interquartile ranges (IQRs) and categorical variables were described as numbers (proportions). The Mann-Whitney U test was applied to compare continuous variables and the χ^2^ test or Fisher’s exact test was used for categorical variables.

Of the 78 deaths, 14 were female (17.9%) and 64 were male (82.1%), with a median age of 50 (IQR, 41-56) years. Forty-four came from Hubei province (56.4%) and 34 from other provinces (43.6%). Twenty-eight (35.9% ) died directly from COVID-19, and the rest (64.1%) died from heart attacks, cerebrovascular accidents, traffic or hotel collapse accidents, fatigue and sudden unexplained death (Table-I).

**Table-I:** Characteristics of healthcare workers who died during the fight against COVID-19 in China.

*Patients, No. (%)*				
Characteristic	Total(n = 78)	Died of COVID-19 infection (n = 28)	Died of other diseases (n = 50)	P value

Age, median (interquartile range), y	50 (41-56)	55 (43-62)	49 (36-52)	0.005
Sex				0.549
Female	14 (17.9)	6 (21.4)	8 (16.0)	
Male	64 (82.1)	22 (78.6)	42 (84.0)	
Professionals^a^				
Yes	7 (9.0)	2 (7.1)	5 (10.0)	0.992
No	71 (91.0)	26 (92.9)	45 (90.0)	
Died in Hubei province				
Yes	44 (56.4)	27 (96.4)	17 (34.0)	< 0.001
No	34 (43.6)	1 (3.6)	33 (66.0)	

a Professionals refer to healthcare workers that worked in emergency departments, infectious diseases departments, respiratory departments, or critical care departments.

Of the 28 healthcare workers who died directly from COVID-19, six were women (21.4%) and 22 were men (78.6%), with a median age of 55 (IQR, 43-62) years. Twenty-seven were from Hubei province (96.4%) and one from Hainan province (3.6%). Fifteen of the healthcare workers (53.6%) were from community or primary hospitals, all but a 59-year-old female nurse were general practitioners (including the one from Hainan province). Four (14.3%) were from secondary hospitals, with specialties in respiratory medicine and intensive care, respiratory medicine, gastroenterology, and traditional Chinese medicine. Nine were from tertiary hospitals (32.1%), including an otolaryngologist, three ophthalmologists and five surgeons (Fig.1).

**Figure:**
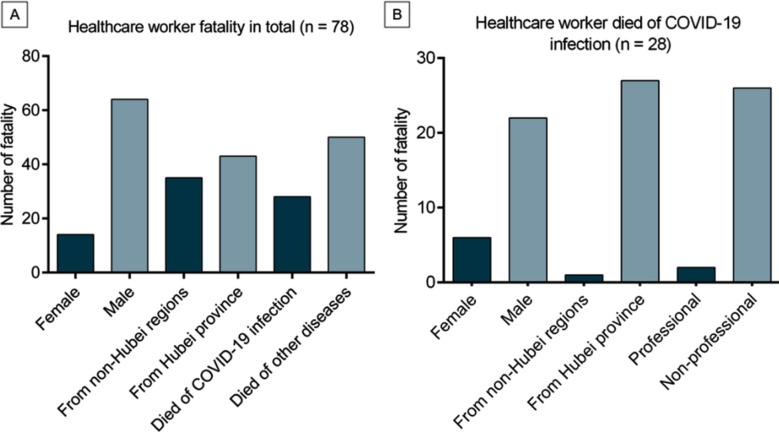
Fig.1

An analysis of the 78 healthcare workers revealed the following characteristics. First, compared to the number of deaths directly attributable to COVID-19, more healthcare workers died from pre-existing disease attack induced by excessive fatigue or died from accidents. This means that people with underlying morbidities such as heart disease and encephalopathy are more at risk when they are overtired or stressed. Second, the median age of the 28 healthcare workers who died directly from COVID-19 in the study was younger than that of the Wuhan 44 non- healthcare workers who died of COVID 19 as reported by Wu et al (55 versus 69 years).^10^ Not only were healthcare workers mostly in employment who were younger than 60 years^11^, but they were also under enormous physical and psychological pressure during the pandemic. Third, although more women than men were involved in fighting the pandemic, more men died. This may be because the female healthcare workers, mostly nurses, were generally younger; the older male healthcare workers may have had more chronic diseases. In addition, the more difficult and dangerous jobs are usually held by men. Fourth, more healthcare workers died in Hubei than in other provinces. This is likely because the outbreak was first and most severe in Hubei province, and the whole society was unprepared and with a critical shortage of personal protective equipment and healthcare workers. Finally, most of the healthcare workers who died directly from COVID-19 were non-professionals. Non-professionals may not have understood the severity of COVID-19 and likely had not received relevant professional training or received appropriate protection measures.

Therefore, rapid and transparent communication is paramount when infectious diseases emerge^12^, which can relieve people’s tension and anxiety. There must be enough healthcare workers who know how to prevent and treat infectious diseases, and they must be provided with adequate personal protective equipment.
